# Polyphyllin VII Promotes Apoptosis and Autophagic Cell Death via ROS-Inhibited AKT Activity, and Sensitizes Glioma Cells to Temozolomide

**DOI:** 10.1155/2019/1805635

**Published:** 2019-11-14

**Authors:** Dejiang Pang, Chao Li, Chengcheng Yang, Yuanfeng Zou, Bin Feng, Lixia Li, Wentao Liu, Yi Geng, Qihui Luo, Zhengli Chen, Chao Huang

**Affiliations:** ^1^Laboratory of Experimental Animal Disease Model, College of Veterinary Medicine, Sichuan Agricultural University, Chengdu 611130, China; ^2^Neuroscience & Metabolism Research, State Key Laboratory of Biotherapy, West China Hospital, Sichuan University and Collaborative Innovation Center, Chengdu 610041, China; ^3^Key Laboratory of Animal Disease and Human Health of Sichuan Province, College of Veterinary Medicine, Sichuan Agricultural University, Chengdu 611130, China; ^4^Natural Medicine Research Center, College of Veterinary Medicine, Sichuan Agricultural University, Chengdu 611130, China; ^5^Animal Nutrition Institute, Sichuan Agricultural University, Chengdu 611130, China

## Abstract

The high recurrence frequency of gliomas but deficiency of effective treatment and prevalent chemoresistance have elicited interests in exploring and developing new agents. Paris polyphyllins are monomers extracted from rhizome of Paris polyphylla var. yunnanensis. Here, we first reported that polyphyllin VII (PP7) exhibited cytotoxic effect on glioma cells. PP7 significantly suppressed the viability and induced cell death of U87-MG and U251 cells after 24 h, with the IC50 values 4.24 ± 0.87 *μ*M and 2.17 ± 0.14 *μ*M, respectively. Both apoptotic and autophagic processes were involved in the cytotoxic effect of PP7, as PP7 activated the Bcl2/Bax pathway and the inhibition of autophagy partly rescued the toxicity of PP7 in glioma cells. In addition, an inhibition of AKT/mTORC1 activity was found after PP7 administration, and it seemed that the overproduction of reactive oxygen species (ROS) was responsible for this effect. Namely, the removal of ROS by NAC treatment mitigated PP7-induced cell death, autophagy, and its effect on the AKT/mTORC1 signaling. Additionally, a combination assay of PP7 with temozolomide (TMZ), the most used chemotherapy for glioma patients, was performed resulting in synergism, while PP7 reduced TMZ resistance through inhibition of MGMT expression. Thus, our study reports PP7 as a potential agent for glioma treatment and reveals its underlying mechanisms of action.

## 1. Introduction

Gliomas are the most common type of primary tumors in the brain, accounting for 30% of all brain and central nervous system tumors and 80% of all the malignant brain tumors [1]. The recurrence and mortality rates of gliomas are quite high, resulting in poor prognosis. Despite progresses in neurosurgery and radio- and chemotherapy, the fatality rate of glioma patients is still high according to recent reports [2, 3]. The median survival time of glioma patients is 12–15 months, the rate of high-grade glioma patients survival over 1 year is lower than 30%, and the 5-year survival rate after diagnosis is 5% [4, 5]. The alkylating agent temozolomide (TMZ) is the most used treatment for glioma after surgical resection and radiotherapy [4]. TMZ methylates DNA at the O^6^ position of guanine, which may cause mismatch pairing of guanines with thymines rather than cytosines resulting in hypermutation and genomic instability that finally leads to cell death [6]. However, the side effects of TMZ were widely reported, such as low white blood cell counts, fever, swollen gums, trouble breathing, and many others according to the Electronic Medicines Compendium (UK) [7, 8]. Previous data showed that TMZ-induced hypermutation was associated with drug resistance [9]. Therefore, mounting efforts need to be done to explore and develop more effective agents for the glioma treatment, or for to define new combined treatments with TMZ to reduce its side effects [10].

Polyphyllins are a class of saponins isolated from the rhizome of a traditional Chinese herb—Paris polyphylla var. yunnanensis (so-called Rhizoma paridis) [11]. Various polyphyllins have been identified by previous studies, including polyphyllin I (PPI), polyphyllin II (PPII), polyphyllin C (PPC), polyphyllin D (PPD), polyphyllin VI (PP6), and polyphyllin VII (PP7), and a wide range of pharmacological effects of these saponins were also reported, such as anti-inflammatory, immunity-enhancing, and especially antitumor activity [12, 13]. It has been demonstrated that PPI suppressed the proliferation rate of tumor cells, such as non-small-cell lung cancer cells, hepatocellular carcinoma cells, ovarian cancer cells, and osteosarcoma cells [14–17]. PPD inhibited growth of breast cancer cells in vitro and decreased the volume of xenografts formed by these cells [18]. Both PPI and PPD were able to increase the apoptosis of glioma cells [19, 20]. The mechanisms underlying the antitumor properties of polyphyllins are diverse, and previous studies have demonstrated that increased ROS production, autophagy, activated cell death processes, and disrupted cell cycle distribution contribute to the polyphyllin mechanisms of action probably in an associated manner [15, 21].

Polyphyllin VII (PP7) is an active pennogenyl saponin with larger molecular weight than other identified polyphyllins. Researchers have recently focused on its bioactivities in cancer treatment, and effective anticancer property of PP7 was found in liver, lung, breast, and colorectal cancer cells [21–24]. However, the anticancer activity of PP7 and its underlying mechanism against gliomas are still unexplored and not well defined. In this work, we aim to investigate the sensitivity of glioma cells to PP7 *in vitro*, evaluate the possibility of its combination with TMZ, and further reveal the molecular mechanisms behind these processes. Our study is aimed at displaying a new perspective for PP7 application in glioma treatment, which could be beneficial for glioma patients.

## 2. Materials and Methods

### 2.1. Reagents

PP7 was purchased from Chengdu Must Bio-Technology Co., Ltd. (Chengdu, China), and the purity of PPI was ≥98%. N-acetyl-L-cysteine (NAC) was purchased from Sigma-Aldrich (Merck KGaA, Darmstadt, Germany). Insulin-like growth factor 1 (IGF-1) and epidermal growth factor (EGF) were purchased from PeproTech (Rocky Hill, USA). 3-methyladenine (3-MA, S2767) and bafilomycin A1 (Baf-A1, S1413) were from Selleck Chemicals (Houston, TX, USA). TMZ was supplied by Tasly Pharmaceutical Co., Ltd. (Tianjin, China). The Cell Counting Kit-8 (CCK-8) was purchased from Dojindo Molecular Technologie (Kumamoto, Japan). Hoechst33342/PI kit, cell mitochondrial isolation kit, and dihydroethidium were supplied by Beyotime Institute of Biotechnology (Haimen, China).

### 2.2. Cell Culture

The glioma cell lines U87-MG and U251 were obtained from the Shanghai Institutes of Biological Sciences, Chinese Academy of Sciences (Shanghai, China). U87-MG and U251 cells were cultured in Dulbecco's modified Eagle's medium (DMEM; HyClone, GE Healthcare Life Sciences, Logan, UT, USA) supplemented with 10% fetal bovine serum (FBS; Gibco; Thermo Fisher Scientific, Inc., Waltham, MA, USA) and 1% penicillin-streptomycin (Invitrogen; Thermo Fisher Scientific, Inc., Waltham, MA, USA). Cells were maintained at 37°C in a humidified atmosphere (5% CO_2_).

### 2.3. Cell Viability Assay

Cell viability was determined by CCK-8 assay as reported before [1]. The cells were seeded with a density of 5 × 10^3^ cells/well in 96-well plates and incubated, at 37°C, in complete medium (DMEM with 10% FBS) with serial dilutions of PP7 for 24 h. CCK-8 was added to each well of the 96-well plate (CCK-8, plus fresh medium at a fixed ratio of 1 : 10, 100 *μ*l/well), followed by further incubation for 1 h. The absorbance was measured at 450 nm using a microplate reader (Thermo Fisher Scientific, Inc.). The half-maximal inhibitory concentration (IC50), defined as the drug dose at which cell growth is inhibited by 50%, was measured using GraphPad Prism software version 5.0 (GraphPad Software, Inc., La Jolla, CA, USA). The formula was used as follows:

(1) $$\begin{array}{c} \text{Cell viability rate }\left(\%\right)=\frac{\text{Absorbance of test sample}-\text{Absorbance of blank}}{\text{Absorbance of control}-\text{Absorbance of blank}}\times 100\%. \end{array}$$

### 2.4. Hoechst 33342/Propidium Iodide (PI) Staining

Hoechst 33342 and propidium iodide (PI) double staining were performed according to the manufacturer's instructions. Briefly, U87-MG or U251 glioma cells were plated in 12-well plates with a density of 5 × 10^4^ cells/well and treated with PP7 at 37°C for 24 h. The cells were washed in PBS three times and incubated in Hoechst33342/propidium iodide (PI) solution (10 *μ*g/ml) for 30 min at 4°C. Finally, fluorescence microscopy was used to observe the PI and Hoechst-positive signals of U87-MG or U251 glioma cells in 15 nonoverlapping fields, and the cell death rate was calculated by the ration of PI-positive cells to Hoechst 33342-positive cells. For each treatment group, ≥1000 cells were analyzed in triplicate.

### 2.5. Reactive Oxygen Species (ROS) Detection

ROS assay kit purchased from Beyotime Institute of Biotechnology was used according to the manufacturer's instructions and previous report [25]. Dihydroethidium was prepared as a 10 mM solution. U251 and U87-MG glioma cells were treated with PP7 at 37°C for 24 h. Then, cells were incubated with 10 *μ*M dihydroethidium (Eth) in DMEM at 37°C for 30 minutes. Finally, fluorescence microscopy was used to observe the ROS production of U251 glioma cells. The fluorescence intensity of Eth was analyzed by Image-Pro Plus software, and then the fluorescence intensity value of each experimental group was compared with the fluorescence intensity value of the control group to get a relative value and quantified by GraphPad Prism software version 5.0 (GraphPad Software, Inc., La Jolla, CA, USA).

### 2.6. Western Blotting Analysis

Western blotting was performed according to standard procedures as reported before [1]. To extract protein from cultured cells was sonicated in lysis buffer (2% SDS with proteinase inhibitors and phosphatase inhibitor). The protein concentration of each extract was measured using the BCA Protein Assay kit (Thermo Scientific Pierce). Equal amounts of protein from each extract were loaded into each lane of a gel and separated by SDS PAGE. The proteins were transferred onto PVDF membranes using standard procedures. The membranes were then blocked with 5% nonfat dry milk in TBST (TBS with 0.1% Tween 20, pH 7.6) for 1 hour at room temperature (RT) and incubated overnight with respected primary antibody at 4°C. After 3 washes with TBST at RT for 10 minutes each wash, the membranes were incubated 1 hour with a 1 : 10,000 dilution of appropriate secondary antibodies diluted in TBST at RT. The membranes were washed another 3 times with TBST at RT for 10 minutes each wash, proteins were then detected with ECL reagent (Thermo Scientific Pierce), and the membranes were exposed to film (Kodak). The antibodies in our experiments include Bax (Abcam, ab232479, 1 : 1000 dilution), Bcl-2 (Abcam, ab32124, 1 : 1000 dilution), caspase-3 (Abcam, ab197202, 1 : 1000 dilution), cleaved caspase-3 (Abcam, ab32042, 1 : 300 dilution), cytochrome C (Abcam, ab133504, 1 : 1000 dilution), prohibitin (Abcam, ab75766, 1 : 2000 dilution), AKT (Cell Signaling Technology, 9272S, 1 : 2000 dilution), pAKT (S473) (Cell Signaling Technology, 4060S, 1 : 2000 dilution), pAKT (T308) (Cell Signaling Technology, 2965S, 1 : 2000 dilution), pS6 (S240/244) (Cell Signaling Technology, 2215S, 1 : 2000 dilution), p4EBP1 (T37/46) (Cell Signaling Technology, 9459, 1 : 1000 dilution), pS6K (T389) (Cell Signaling Technology, 9205, 1 : 1000 dilution), SQSTM1/P62 (Abcam, ab91526, 1 : 2000 dilution), LC3B (Abcam, ab192890, 1 : 500 dilution), GAPDH (Millipore, MAB374, 1 : 2000 dilution), *β*-actin (Boster,BM0627, 1 : 1000 dilution), Goat anti-Rabbit IgG (H+L) Secondary Antibody (Pierce, 31460 1 : 10,000 dilution), and Goat anti-Mouse IgG (H+L) Secondary Antibody (Pierce, 31430 1 : 10,000 dilution). We used ImageJ software to quantitate western blots. Firstly, we measured the grey value of blots by using ImageJ. Secondly, normalized each sample using a normalizer (housekeeping protein such as GAPDH or *β*-actin, as mentioned above) and then the normalized value of each experimental group was compared with the normalized value of the control group to get a relative value. Finally, we used GraphPad software to make a histogram.

### 2.7. Cell Mitochondria Isolation

We used a cell mitochondrial isolation kit (Beyotime Biotechnology, C3601) for mitochondrial isolation of U87-MG cells and U251 cells. Firstly, we washed the cells with PBS, cells were harvested with trypsin-EDTA solution, centrifuged at 100-200 g for 5-10 minutes at room temperature, and the cells were collected. Secondly, we added 1-2.5 ml mitochondrial separation reagent per 20-50 million cells and then cells were gently suspended and placed on ice for 10-15 minutes. Thirdly, the cell suspension was transferred to a glass homogenizer of appropriate size, homogenizing the cells about 10-30 times. Fourthly, after homogenization, the cell suspension was centrifuged for 10 min at 600g at 4°C. Fifthly, the supernatant was carefully transferred to a new tube and centrifuged for 10 min at 11,000g at 4°C. The precipitate was the isolated mitochondria of the cells. Next, the supernatant was carefully transferred to a new tube and centrifuged for 10 min at 12,000 g at 4°C. The supernatant was the cytoplasmic protein that removed mitochondria. Both cytoplasmic protein and mitochondria were finally lysed for WB detection.

### 2.8. GFP-LC3 Puncta Imaging

We first constructed the GFP-LC3 vector. The cDNA encoding human LC3B (Gene ID: 81631) was obtained by PCR from U251 cDNA. Primer sequences used were as follows: LC3B forward, 5′-GCG TCG ACCA TGC CGT CGG AGA AGAC-3′ and reverse, 5′-GCG CGG CCG CTT ACA CTG ACA ATT TCAT-3′. The forward primers included Sal1 restriction endonuclease restriction site and the reverse primers included Not1 restriction endonuclease restriction site. Firstly, using U251 cDNA as template, LC3B coding sequences were cloned by adding Taq2x mix enzyme and a pair of primers in the PCR amplification system. Secondly, the PCR products were subjected to nucleic acid electrophoresis and LC3B cDNA were purified from the nucleic acid gel. Thirdly, LC3B cDNA and pEGFP-C1 vector were subjected to Sal1/Not1 double restriction enzyme digestion, and then the LC3B cDNA were inserted into the pEGFP-C1 vector by T4 DNA ligase. Fourthly, the ligation products of the previous step were transformed into *E. coli* competent cells, and then the clone was selected and the plasmid was extracted to obtain the GFP-LC3 plasmid. Finally, sequencing was performed to identify the plasmid.

U251 cells and U87-MG cells were seeded with a density of 2 × 10^5^ cells/well in 12-well plates and incubated in complete medium (DMEM with 10% FBS) for overnight and then were transfected with GFP-LC3 plasmid using Lipofectamine™ 2000 (Invitrogen). Briefly, for each well of 12-well plates, we diluted 2 *μ*g GFP-LC3 plasmid into 50 *μ*l medium without serum in tube A and diluted 2 *μ*l of Lipofectamine™ 2000 into 50 *μ*l medium without serum in tube B, and incubated for 5 min at room temperature. Then, we added 50 *μ*l of the tube B to tube A, mixed gently, and incubated at room temperature for 20 min to allow DNA-Lipofectamine™ 2000 complexes to form. This DNA-Lipofectamine™ 2000 complex (100 *μ*l) was added directly into each well of the plates containing cells and mixed gently. Twenty-four hours after transfection, corresponding treatments were performed for 8 h. Finally, fluorescence microscopy was preformed to observe the GFP-LC3 puncta of U251 glioma cells as described before [26]. The number of GFP-LC3B puncta per cells was assessed in 10 nonoverlapping fields and measured by Image-Pro Plus software, and then we calculated the average LC3 puncta per cells in these 10 nonoverlapping fields and made the graph by GraphPad Prism software. For the treatment of 3-MA, U251 and U87-MG cells were treated with PP7 (2 *μ*M and 4 *μ*M to U251 cells, 3 *μ*M and 6 *μ*M to U87-MG cells) for 8 h, 3-MA (5 mM) was added into U251 and U87-MG cells 3 hours after adding PP7, and then the puncta were evaluated. In CCK-8 assay, U251 and U87-MG cells were treated with PP7 (4 *μ*M to U251 cells, 6 *μ*M to U87-MG cells) alone or in combination with 3-MA (5 mM) for 24 h. For the treatment of bafilomycin A1, U251 and U87-MG cells were treated with PP7 (4 *μ*M to U251 cells, 6 *μ*M to U87-MG cells) alone or together with bafilomycin A1 (10 nM) for 24 h.

### 2.9. Real-Time PCR

Total RNA was extracted from the U251 cells and U87-MG cells using TRIzol reagent (Invitrogen; Thermo Fisher Scientific, Inc.), according to the manufacturer's instructions after being treated with indicated concentrations of PP7 or TMZ. In each group, 2 *μ*g RNA was reverse transcribed into cDNA using the Revert Aid First Strand cDNA Synthesis kit (Thermo Fisher Scientific, Inc.) for RT-PCR as described before [1]. One microliter cDNA was added to a total volume of 20 *μ*l. PCR amplification system qPCR was performed using the SYBR Premix Ex Taq™ II kit (Takara Biotechnology, Co., Ltd., Dalian, China). Relative fold levels were determined using GAPDH gene as normalizer control. The qPCR results were analyzed by Bio-Rad CFX Manager 3.0 software, and the normalized value of each experimental group was compared with the normalized value of the control group to get a relative value. Threshold cycle (Ct) values should be within the range mean ± 1 for each reference gene across all samples to ensure similar cDNA yield from each RT reaction. Primer sequences used were as follows: MGMT mRNA forward, 5′- ACC GTT TGC GAC TTG GTA CTT-3′ and reverse, 5′- GGA GCT TTA TTT CGT GCA GACC-3′. GAPDH mRNA forward, 5′- GGA GCG AGA TCC CTC CAA AAT -3′ and reverse, 5′- GGC TGT TGT CAT ACT TCT CAT GG -3′.

### 2.10. Statistical Analysis

Statistical evaluation was conducted using SPSS 19.0 (SPSS, Inc., Chicago, IL, USA) for Windows package software. Data from western blots, LC3 punctum quantifications are presented as the mean ± standard deviation, while the others are presented as mean ± standard error of mean. Differences among multiple groups were compared by one-way analysis of variance, and differences between two groups were compared by the Student *t*-test. *p* < 0.05 was considered to indicate a statistically significant difference.

## 3. Results

### 3.1. PP7 Decreases the Viability of U87-MG and U251 Cells

**Figure 1:**
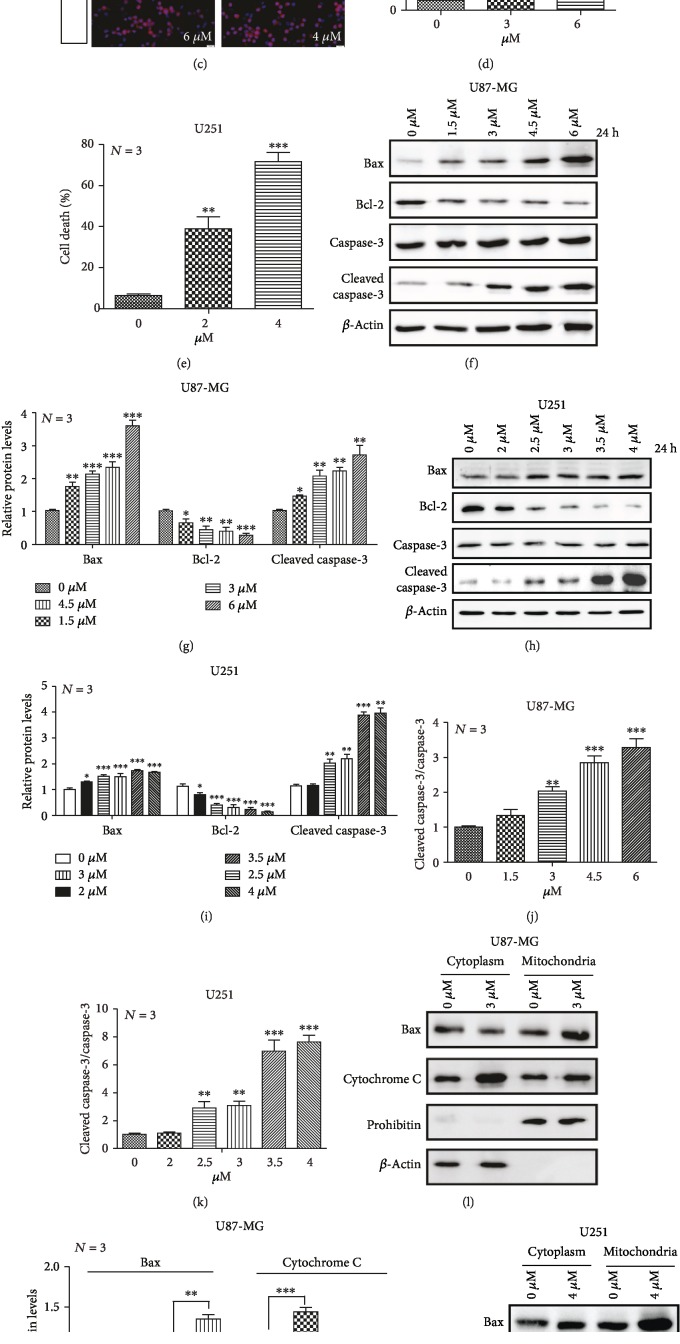
PP7 cytotoxicity in U87-MG and U251 cells. (a, b) The cell viability of U87-MG and U251 cells treated with different concentrations of PP7 was assessed after 12, 24, and 36 h. (c–e) Representative images and quantifications display increased cell death of U87-MG and U251 cells under PP7 treatment for 24 h, labeled with Hoechst33342/PI. Arrows indicate dead cells. (f, g) Western blots and their quantification show increased Bax and cleaved caspase-3 protein levels but decreased Bcl-2 levels in a dose-dependent manner in U87-MG cells and (h, i) in U251 cells. (j, k) Quantifications of the ratio of cleaved caspase-3 to total caspase-3 in U87-MG and U251 cells after PP7 treatment. (l, m) Western blots and their quantification show increased Bax protein levels in mitochondria as well as increased cytochrome C protein levels in cytoplasm after PP7 treatment in U87-MG cells and (n, o) in U251 cells. Prohibitin was selected as loading control for mitochondrial fraction, while *β*-actin was an internal control for the cytoplasmic fraction. Solvent controls are presented as 0 *μ*M groups, while *N* stands for the repetition of experiments. ^∗^*p* < 0.05, ^∗∗^*p* < 0.01, ^∗∗∗^*p* < 0.001.

To evaluate the cytotoxic effect of PP7, two human glioma cell lines (U87-MG and U251) were exposed to PP7 at different concentrations for 12, 24, and 36 h before CCK-8 assay. As shown in Figures 1(a) and 1(b), cell viability of both U87-MG and U251 cells was suppressed by PP7, while the most pronounced dose-dependent effect was achieved after 24 h with IC50 values 4.24 *μ*M ± 0.87 *μ*M and 2.17 ± 0.14 *μ*M, respectively. According to these IC50 values, concentrations of 3 *μ*M and 6 *μ*M PP7 for U87-MG cells, as well as 2 *μ*M and 4 *μ*M PP7 for U251 cells, were chosen for most of the following experiments. The decrease in cell viability of U87-MG and U251 cells after PP7 treatment resulted from cell death induction according to the experiments performed by Hoechst33342/propidium iodide (PI) double staining (Figures 1(c)–1(e)). To discriminate the cell death type, we analyzed the expression of Bcl-2 family proteins as well as cleaved caspase 3 and found that PP7 reduced the protein levels of Bcl-2, which was accompanied with an increase of Bax and cleaved caspase-3 levels in U87-MG and U251 cells (Figures 1(f)–1(k)). In addition, the sublocalizations of Bax and cytochrome C were compared between mitochondria and cytoplasm, showing significant enrichment of Bax in mitochondria while cytochrome C was more abundant in cytoplasm fraction (Figures 1(l)–1(o)). All these results demonstrated that PP7 decreased the viability of glioma cells is mediated by the induction of apoptosis.

### 3.2. PP7 Promotes Reactive Oxygen Species (ROS) Production in U87-MG and U251 Cells

**Figure 2:**
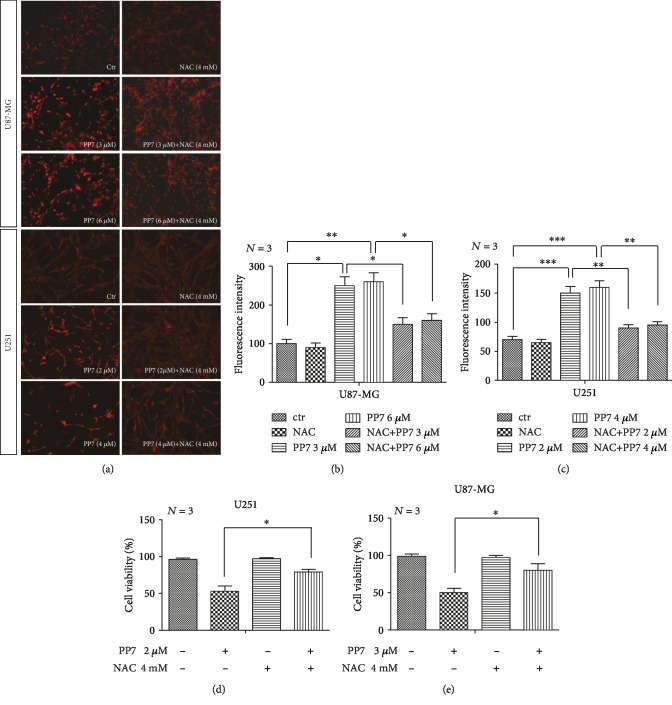
PP7 promotes ROS production in U87-MG and U251 cells. (a–c) Representative images and quantification analysis of PP7 effect on ROS production in U87-MG and U251 cells, assessed by dihydroethidium labeling (a, left) and clearance of ROS after NAC treatment (a, right). (d, e) Quantification of CCK-8 assay shows that NAC administration increases cell viability of PP7-treated U251 and U87-MG cells. Ctr represents cells treated with solvent, while *N* stands for the repetition of experiments. ^∗^*p* < 0.05, ^∗∗^*p* < 0.01, ^∗∗∗^*p* < 0.001.

Potential anticancer compounds able to promote ROS production in cancer cells have a good prospect for further preclinical investigations. In our study, we found significantly increased ROS accumulation in U87-MG and U251 cells after PP7 treatment, which was measured by fluorescent dihydroethidium (Eth) labeling (Figures 2(a) left, 2(b), and 2(c)). To study the relationship between ROS production and cytotoxic effect induced by PP7, we further performed ROS clearance with the common antioxidant N-acetylcysteine (NAC). As shown by Eth labeling, ROS accumulation was decreased after NAC treatment (Figures 2(a) right, 2(b), and 2(c)). In addition, significantly increased cell viability was detected by CCK-8 assay in U87-MG and U251 cells exposed to NAC/PP7 combined treatment (Figures 2(d) and 2(e)). These results indicated that overproduction of ROS was involved in PP7 cytotoxicity of glioma cells.

### 3.3. ROS Generated from PP7 Treatment Induces Autophagy in U87-MG and U251 Cells

**Figure 3:**
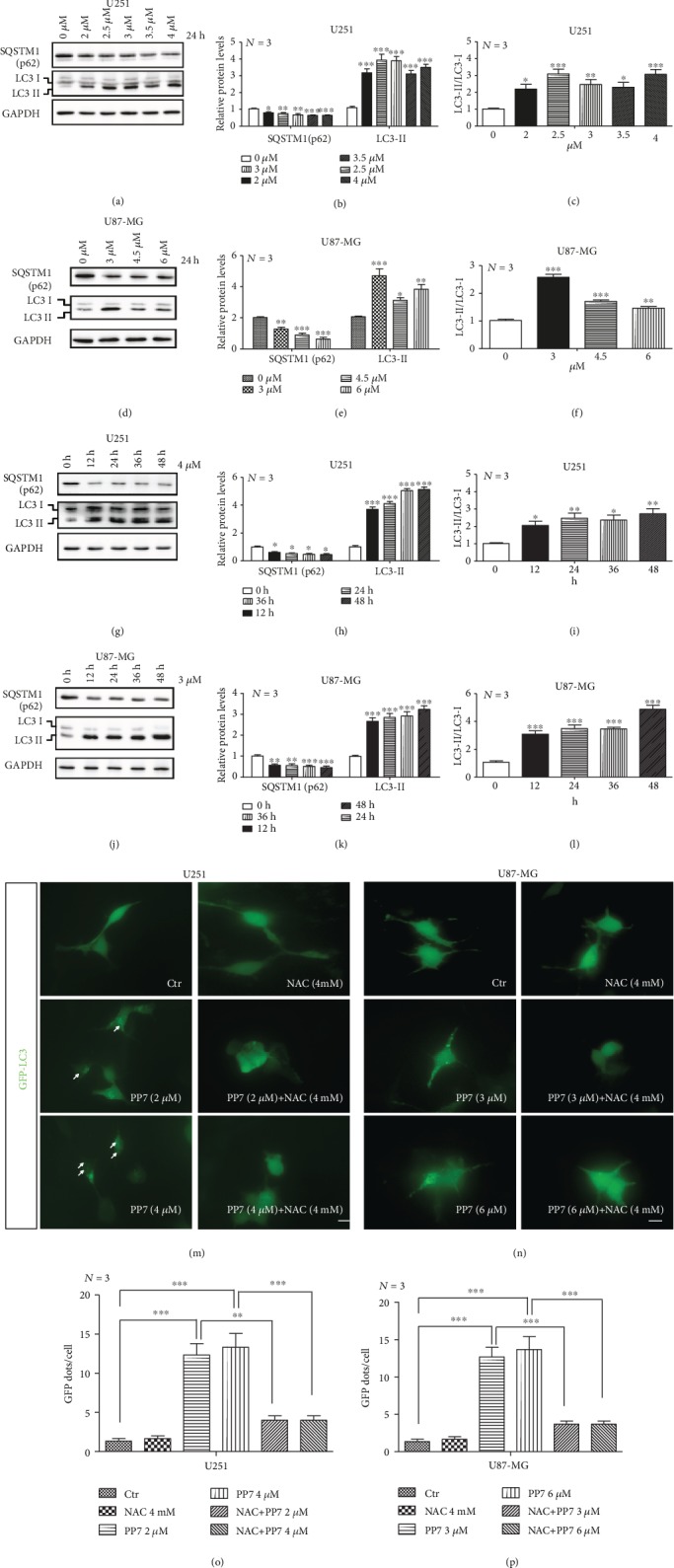
PP7 induces autophagy in U87-MG and U251 cells. (a–c) Western blots and their quantification show PP7 concentration-dependent decreased SQSTM1 (p62) protein levels and increased LC3II levels accompanied with the increase in LC3 II/LC3 I ratio in U251 cells as well as (d–f) in U87-MG cells. Solvent-treated cells are presented as the 0 *μ*M control groups. (g–i) Western blots and their quantification show time-dependent decreased SQSTM1 (p62) protein levels and increased LC3II levels accompanied with the increase in LC3 II/LC3 I ratio in U251 cells treated with 4 *μ*M PP7 as well as (j–l) in U87-MG cells treated with 3 *μ*M PP7. (m–p) Representative images and quantification analysis display that PP7 promotes the formation of GFP-LC3 puncta in U251 and U87-MG cells, and this process could be inhibited by NAC treatment. Ctr stands for the cells treated with solvent. Arrows indicate the LC3 puncta. *N* stands for the repetition of experiments. ^∗^*p* < 0.05, ^∗∗^*p* < 0.01, ^∗∗∗^*p* < 0.001.

To investigate whether the overproduction of ROS in PP7-treated glioma cells induced cellular autophagy, the protein levels of widely used autophagy markers—LC3 and SQSTM1 (p62)—were analyzed. In our study, SQSTM1 (p62) protein levels were significantly reduced, while increased LC3 II/LC3 I ratio was observed in U251 and U87-MG cells under a series of PP7 increasing concentrations and at different time points (Figures 3(a)–3(l)). To further corroborate this finding, GFP-LC3 plasmids were transfected into U251 and U87-MG cells. We observed large amounts of fluorescent puncta formed in the cytoplasm of U87-MG and U251 cells after PP7 treatment, displaying the presence of LC3 conjugation that is considered as a hallmark event in the autophagic process (Figures 3(m) left and 3(n) left). These results indicated that PP7 indeed induces autophagy in glioma cells. To investigate the role of ROS in PP7-induced autophagy, we further performed the ROS clearance experiment with the administration of NAC. We found that the formation of GFP-LC3 puncta induced by PP7 could be easily suppressed by the treatment of NAC, suggesting that the PP7-stimulated ROS overproduction was implicated in the subsequent autophagic process (Figures 3(m) right, 3(n) right, 3(o), and 3(p)).

### 3.4. Autophagy Contributes to PP7 Cytotoxic Effect in Glioma Cells

**Figure 4:**
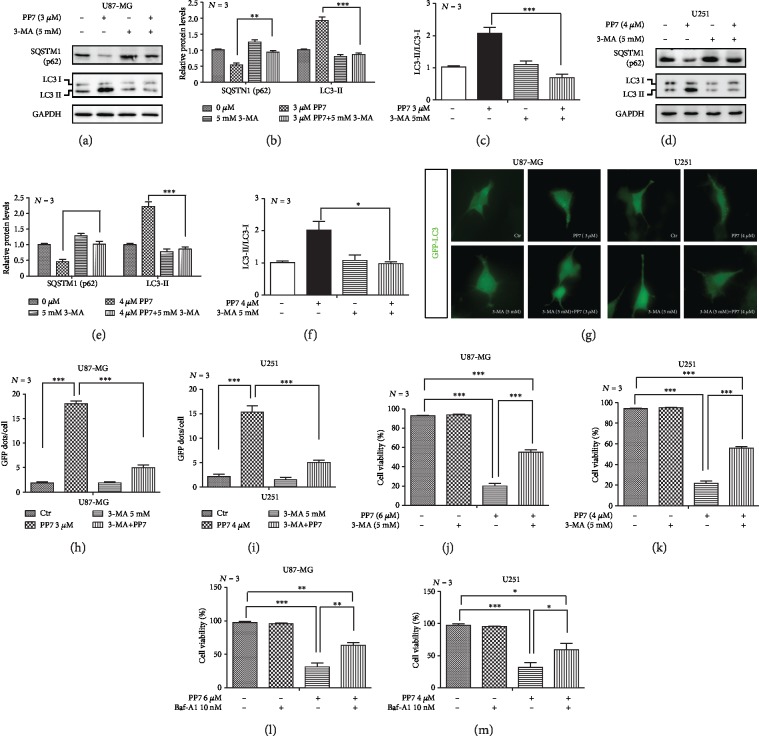
Autophagy is involved in the antiglioma effect of PP7. (a–c) Western blots and corresponding quantification analysis show the effect of 3-MA in respect to increased SQSTM1 (p62), decreased LC3II protein levels, and the decrease in LC3 II/LC3 I ratio in U87-MG cells and (d–f) in U251 cells. U87-MG and U251 cells were subsequently treated with 3 *μ*M PP7 and 3 *μ*M PP7, respectively, and with 5 mM 3-MA. 3-MA was added 3 h after adding PP7, while the overall treatment lasted 8 h. (g–i) Representative images and quantification analysis display decrease in LC3 puncta after PP7 and 3-MA combined treatment in U251 and U87-MG cells. Ctr represents cells treated only with solvent. (j, k) Cytotoxic effect induced by PP7 was partly rescued by 3-MA treatment in U87-MG and U251 cells. U87-MG and U251 cells were treated with 6 *μ*M and 4 *μ*M PP7, respectively, alone or in combination with 3-MA (5 mM) for 24 h. (l, m) Cytotoxic effect induced by PP7 was partly rescued by Baf-A1 treatment in U87-MG and U251 cells. U251 and U87-MG cells were treated with 4 *μ*M and 6 *μ*M PP7, respectively, alone or in combination with bafilomycin A1 (10 nM) for 24 h. *N* stands for the repetition of experiments. ^∗^*p* < 0.05, ^∗∗^*p* < 0.01, ^∗∗∗^*p* < 0.001.

To evaluate whether autophagy was also implicated in PP7-suppressed glioma cells viability, 3-methyladenine (3-MA) was applied as an autophagy inhibitor. We found that PP7-induced autophagy could be inhibited by 3-MA both in U87-MG and U251 cells, as shown by increased SQSTM1 (p62), decreased LC3II protein levels, and the decrease in LC3 II/LC3 I ratio (Figures 4(a)–4(f)). Moreover, significantly less LC3 puncta were observed in both glioma cell lines exposed to PP7 and 3-MA combined treatment (Figures 4(g)–4(i)). Most importantly, cytotoxicity induced by PP7 was partly rescued by 3-MA treatment in U87-MG and U251 cells (Figures 4(j) and 4(k)). To strengthen our observations, we further performed the experiments with another widely used autophagy inhibitor—bafilomycin A1 (Baf-A1) in U87-MG and U251 cells. The results showed that PP7 cytotoxic effect was also partly rescued by bafilomycin A1 treatment (Figures 4(l) and 4(m)). Therefore, the autophagic cell death, along with apoptosis, is involved in the antiglioma effect of PP7.

### 3.5. PP7 Inhibits the AKT/mTORC1 Signaling Pathway in U251 and U87-MG Cells

**Figure 5:**
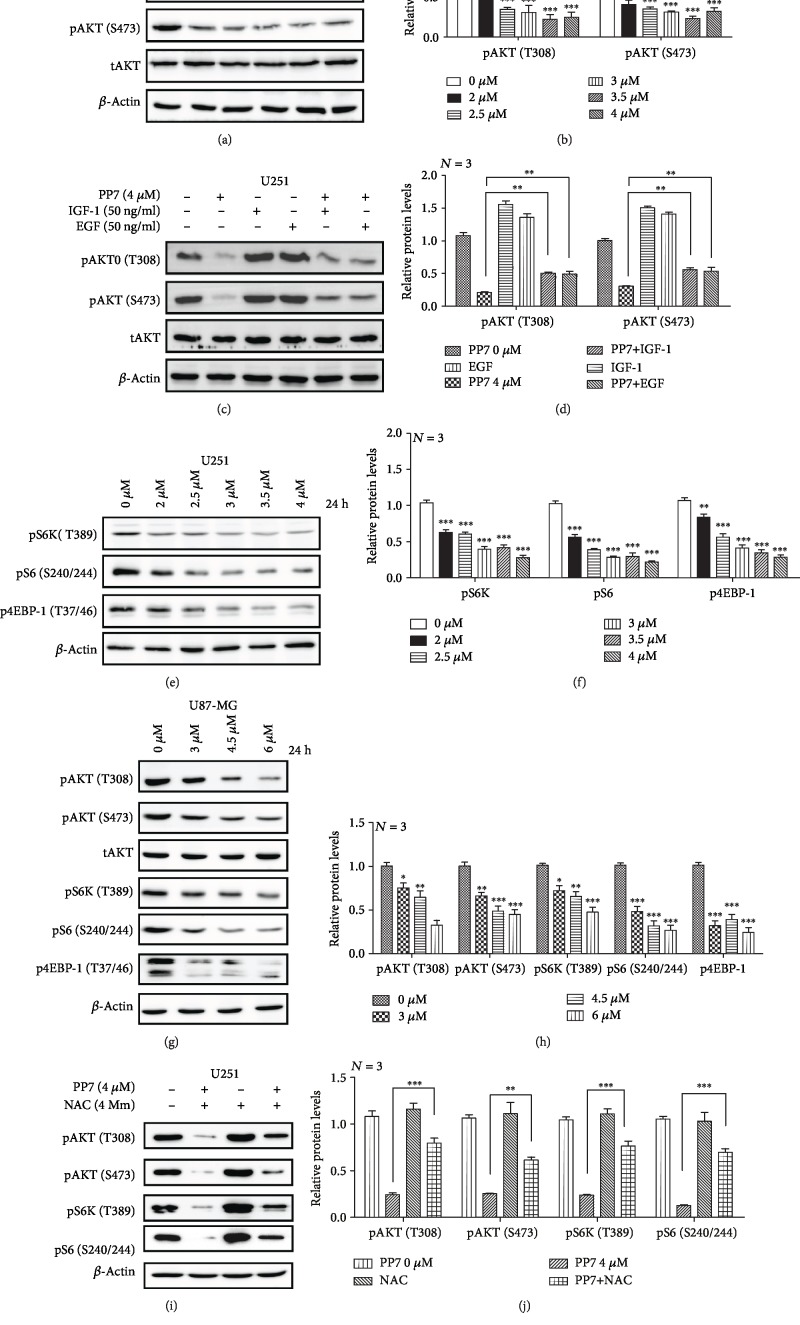
PP7 inhibits the AKT/mTORC1 pathway in U251 and U87-MG cells. (a, b) Western blots and corresponding quantification analysis show decreased phosphorylation levels of AKT in PP7-treated U251cells. Solvent-treated cells are presented as 0 *μ*M control. (c, d) Western blots and corresponding quantification analysis show the effects of growth factors' application on phosphorylation levels of AKT after PP7 treatment in U251cells. (e, f) Western blots and corresponding quantification analysis show decreased phosphorylation levels of mTORC1 effectors, 4E-BP1, p70S6K, and S6, in U251 cells treated with PP7. (g, h) Western blots and corresponding quantification analysis show that PP7 inhibits the AKT/mTORC1 pathway in U87-MG. Solvent-treated cells are represented as 0 *μ*M control. (i, j) Western blots and corresponding quantification show that the NAC administration recovers the phosphorylation levels of AKT and mTORC1 effectors induced by PP7 treatment in U251cells. *N* stands for the repetition of experiments. ^∗∗^*p* < 0.01, ^∗∗∗^*p* < 0.001.

Taking into consideration that AKT promotes antiapoptotic and protumorigenic activities, we investigated the effect of PP7 on AKT activation. The treatment of U251 cells with PP7 resulted in a notable decreased phosphorylation levels of AKT, while the total levels of AKT remained unchanged, which suggested that PP7 suppresses the activity of AKT pathway (Figures 5(a) and 5(b)). Furthermore, we showed that suppressed AKT phosphorylation could be rescued by growth factors (IGF-1 and EGF) (Figures 5(c) and 5(d)). AKT signaling is involved in the regulation of mTORC1 activity; thus, the effect of PP7 on mTORC1 activity was further analyzed. We found that suppressed AKT activity after PP7 treatment caused the inhibition of the mTORC1 complex activity, as illustrated by the decreased phosphorylation levels of mTORC1 effectors, 4E-BP1, pS6K, and S6 (Figures 5(e) and 5(f)). In addition, we further evaluated the effects of PP7 on AKT/mTORC1 signaling in U87-MG cells and found the same pattern as in U251 cells (Figures 5(g) and 5(h)). In order to investigate whether the inhibitory response on AKT/mTORC1 signaling was also dependent on ROS, NAC was administrated in combination with PP7, resulting in recovered phosphorylation levels of both mTORC1 effectors and AKT (Figures 5(i) and 5(j)). Therefore, our work demonstrated that AKT/mTORC1 signaling pathway is involved in PP7 antiglioma effect.

### 3.6. Synergistic Cytotoxic Effect of PP7 and TMZ Combination in U251 and U87-MG Cells

**Figure 6:**
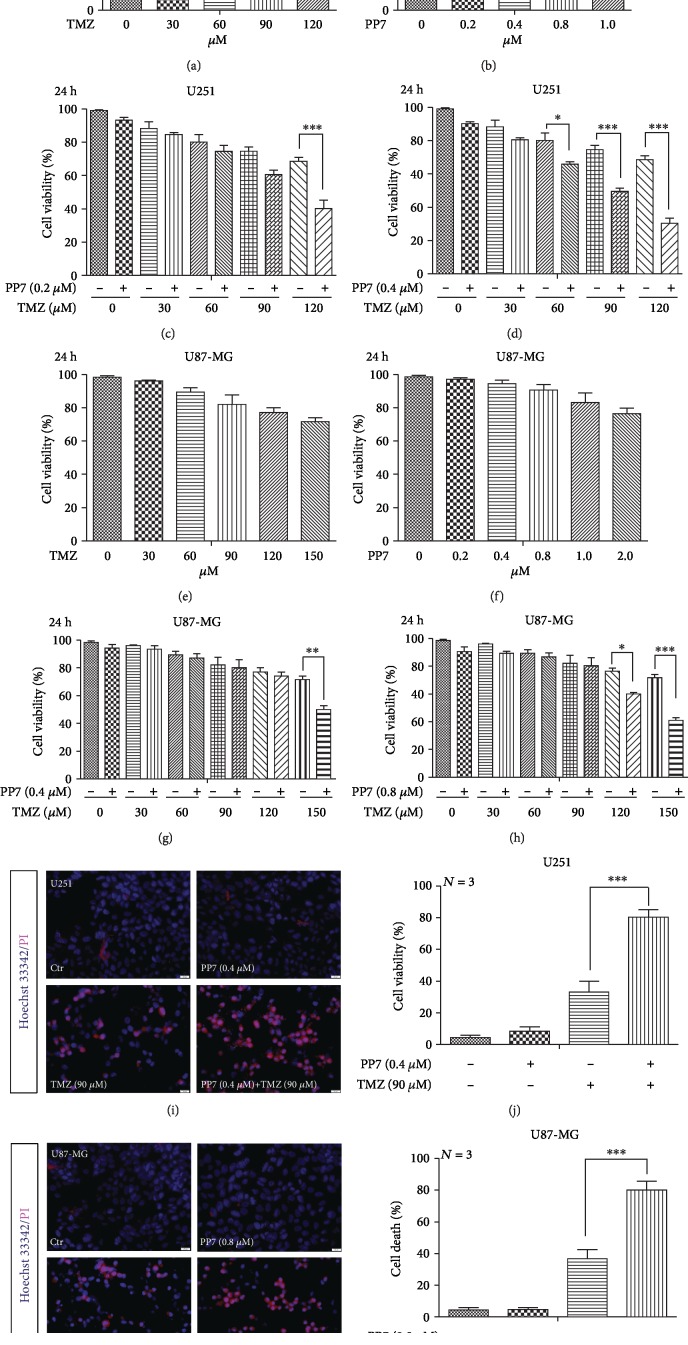
Synergistic cytotoxic effect of PP7 and TMZ combination in U251 and U87-MG cells. (a, b) Cell viability of U251 cells treated with ineffective concentrations of PP7 or TMZ separately for 24 h (*N* = 3). (c, d) Cell viability of U251 cells treated with combinations of PP7 and TMZ for 24 h (*N* = 4). (e, f) Cell viability of U87-MG cells treated with ineffective concentrations of PP7 or TMZ separately for 24 h (*N* = 3). (g, h) Cell viability of U87-MG cells treated with combinations of PP7 and TMZ for 24 h (*N* = 4). (i, j) Representative images and quantification analysis show that low concentration of PP7, combined with TMZ, induced cell death in U251 cells. Solvent controls are represented as 0 *μ*M. ^∗^*p* < 0.05, ^∗∗∗^*p* < 0.001. (k, l) Representative images and quantification analysis show that low concentration of PP7, combined with TMZ, induced cell death in U87-MG cells. Solvent controls are represented as 0 *μ*M. *N* stands for the repetition of experiments. ^∗^*p* < 0.05, ^∗∗∗^*p* < 0.001.

Temozolomide (TMZ) is the main chemotherapy utilized for glioma treatment with serious side effects. Therefore, the agents that could secure the efficacy of TMZ when applied in combination and reduce its side effects due to the decreased effective dose will be valuable for glioma treatment. In order to evaluate the potential of PP7 in glioma treatment combined with TMZ, low ineffective concentrations of PP7 (0.2 *μ*M and 0.4 *μ*M for U251, 0.4 *μ*M and 0.8 *μ*M for U87-MG) were combined with different concentration gradients of TMZ, which displayed no statistically significant cytotoxicity to U251 and U87-MG cells (Figures 6(a), 6(b), 6(e), and 6(f)). The combined treatments lasted 24 h. We found that the combination of PP7 and TMZ significantly increased the cytotoxicity in U251 and U87-MG cells, especially when 0.4 *μ*M PP7 were applied in combination with TMZ in U251 cells (Figures 6(c) and 6(d)) and 0.8 *μ*M in U87-MG cells (Figures 6(g) and 6(h)). Besides, Hoechst 33342/PI staining confirmed the synergy of PP7 and TMZ in glioma cell death induction (0.4 *μ*M PP7 with 90 *μ*M for U251 and 0.8 *μ*M PP7 with 150 *μ*M for U87-MG) (Figures 6(i)–6(l)). These data indicated that even low concentrations of PP7 can increase the cytotoxicity of TMZ, which offers a broad application prospect for PP7.

### 3.7. PP7 Reduces TMZ Resistance in U251 and U87-MG Cells by Suppressing the Expression of MGMT

**Figure 7:**
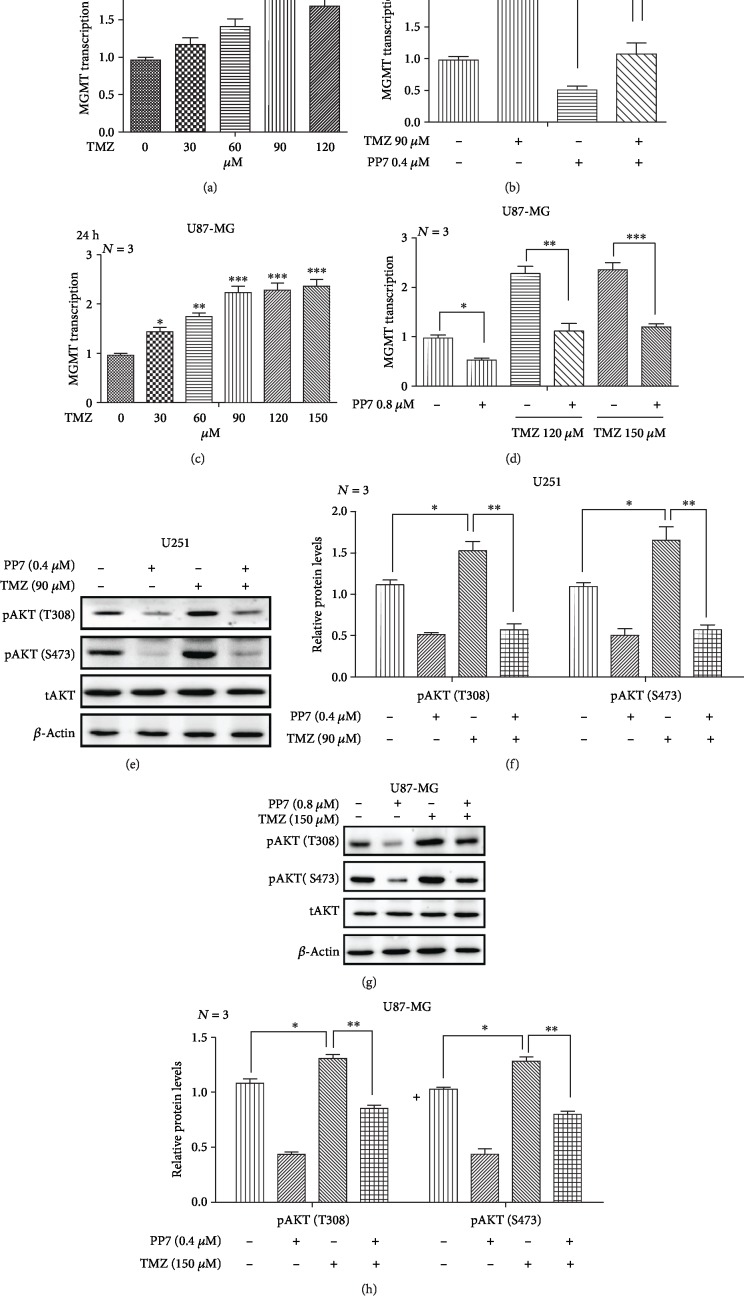
PP7 reduces TMZ resistance in U251 and U87-MG cells by suppressing the expression of MGMT. (a) Quantified mRNA expression of MGMT after TMZ treatment in U251 cells (*N* = 3). (b) PP7 suppresses the expression of MGMT induced by TMZ treatment in U251 cells (*N* = 3). (c) Quantified mRNA expression of MGMT after TMZ treatment in U87-MG cells (*N* = 3). (d) PP7 suppresses the expression of MGMT induced by TMZ treatment in U87-MG cells (*N* = 3). (e, f) Western blots and corresponding quantification analysis show that TMZ activates the phosphorylation of AKT, which could be inhibited by PP7 in U251 cells (*N* = 3). (g, h) Western blots and corresponding quantification show that TMZ activates the phosphorylation of AKT, which could be inhibited by PP7 in U87-MG cells (*N* = 3). Solvent controls are represented as 0 *μ*M. *N* stands for the repetition of experiments. ^∗^*p* < 0.05, ^∗∗^*p* < 0.01, ^∗∗∗^*p* < 0.001.

In addition to the side effects, TMZ resistance resulting from abnormal expression of MGMT is also an important factor affecting the treatment of gliomas. In our study, we found the induction of MGMT expression in U251 and U87-MG cells after TMZ treatment, and this induction could be suppressed by the combined treatment of PP7 with TMZ (Figures 7(a)–7(d)). As the AKT pathway was proven to be involved in the transcriptional regulation of MGMT, we further investigated the effects of PP7 on AKT phosphorylation which was increased by TMZ treatment in U251 and U87-MG cells (Figures 7(e)–7(h)). These results suggested that drug resistance will be suppressed if PP7 is applied in combination with TMZ.

## 4. Discussions

PP7 has been reported to exhibit strong anticancer activities in some cancer types. Zhang et al. reported that PP7 induced HepG2 cells death through an inhibition of the PI3K/AKT/mTOR pathway [22], while Lin et al. found an induction of G2/M cell cycle arrest and apoptosis in PP7-treated lung cancer cells [21]. In glioma cells, polyphyllin D (PPD) has been reported to induce apoptosis in U87 cells through the JNK pathway [20], and PP1 has been reported to induce cell cycle arrest and apoptosis in U251 cells also via the JNK pathway [19]. However, the anticancer activity of PP7 in glioma cells and its mechanism have not been defined. In this present work, we displayed that PP7-induced ROS production suppressed the activity of AKT signaling to induce cell death in U251 and U87-MG cells. Apoptosis is one of the major cell death-related pathways, and Bcl-2 family is responsible for the release of proapoptotic factors, such as cytochrome C, to trigger mitochondria-related and caspase-3 dependent apoptosis [27]. Antiapoptotic Bcl-2 proteins (e.g., Bcl-2 and BCL-xL) function to directly bind and inhibit the proapoptotic BCL-2 proteins (e.g., Bax) [28]. Autophagy is a regulated lysosomal pathway critical for the long-lived proteins and organelle degradation and recycling [29]. The roles of autophagy in cancers are complex, and controversial studies were published [30, 31]. However, lots of works supported an anticancer property for autophagy, as the autophagy gene Beclin-1 was proven to be a tumor suppressor, while the tumor suppressor genes p53 and PTEN were demonstrated to induce autophagy [32–34]. In our study, we found increased Bax levels, cytochrome C release, and induction of autophagy in PP7-treated glioma cells, which suggested that PP7-induced glioma cells death may be resulted from increased apoptosis and autophagic cell death. Meanwhile, the U251 cells were more sensitive to PP7 than U87-MG cells, which may be due to different expressions of critical genes between them. For example, U87-MG express WT p53, while U251 contained mutant p53, and the expression of glutathione S-transferase omega-1, a member of glutathione S-transferase super family that is implicated in the drug resistance of cancer cells, is higher in U87-MG cells than that in U251 cells [35, 36].

Physiologically, ROS are normal by-products generated from metabolic or enzymatic processes [1]. Compelling evidences have indicated that ROS function as a critical regulator not only in normal cells but also in many cancer cells for multiple cellular signaling pathways [1]. In order to keep high proliferation metabolic rates, cancer cells maintain much more ROS than normal ones. Although numerous physiological roles of ROS have been reported, especially as signaling molecules involved in cell growth, differentiation, and tumor progression. The redox balance in cancer cells is of critical importance, and excessive ROS accumulations are catastrophic for cancer cells [37]. Besides, the antitumor property of ROS has elicited interests in recent years. Researchers have reported that ROS were implicated in selective killing of cancer cells [38], but a clear understanding of the mechanisms underlying these processes is still elusive. The AKT/mTOR pathway plays a crucial role in regulating cellular proliferation, cell cycle, survival, and metabolism [39]. The impact of ROS on AKT/mTOR was widely investigated, yielding conflicting results. Some studies reported the amplification of AKT/mTOR activity by ROS through an inactivation of PTEN [40–42], whereas the others found that ROS were capable of modulating AKT itself with an inhibitory effect, especially under high doses [43–46]. In our study, we found the phosphorylation of AKT and mTORC1 downstream effectors were dramatically downregulated by PP7 administration, and this response could be reversed by NAC treatment. These results were in concert with the recent publications regarding negative redox regulation of AKT/mTORC1 and provided evidence that PP7 mediated AKT/mTORC1 inhibition via an ROS-dependent mechanism [43–46].

The relationship between autophagy and tumorigenesis has been well established. Autophagy could provide cancer cells with additional energy or nutrients in some specific periods, and it also functions as a cell death and tumor suppressor mechanism [47]. Although physiological level of autophagy is essential for cellular homeostasis maintenance under various stress conditions, excessive or uncontrolled level of that is able to induce autophagic cell death [30]. Furthermore, as different classifications of cell death, apoptosis and autophagic cell death display intricate interrelationships between each other. Under some conditions, synergetic effects of apoptosis and autophagy could be found, whereas in other situations, autophagy can be triggered only when apoptosis is suppressed, indicating that different types of cell death may develop concomitantly to response to different stress conditions [47, 48]. Meanwhile, the relationship between ROS accumulation and autophagy has been widely studied in the recent years, and it is believed that ROS are an important inducer of autophagy [29]. In our study, we found that PP7 could induce both apoptosis and autophagy in glioma cells, which was dependent on the generation of ROS. In addition to the coordination of energy, and growth signals, mTORC1 has been proven to modulate cellular autophagy [49]. Expectedly, the suppressed mTORC1 activity resulted in an induction of autophagy in glioma cells, as demonstrated by the accumulation of LC3 and decreased p62 levels in PP7-treated cells. Moreover, the LC3 accumulation was further proven by detection of autophagic puncta in GFP-LC3 transfected glioma cells treated with PP7. Our study also found that the cell death present in PP7-treated glioma cells could be partly rescued by 3-MA and Baf-A1, inhibitors of autophagy [50, 51], thus providing clear evidences for the autophagy role in PP7-induced glioma cell death.

Surgical resection, radiotherapy, and TMZ chemotherapy are the standard therapy for glioma patients, but seriously, side effects [52] and the resistance to TMZ due to the expression of MGMT resulted in limiting efficacy of this treatment. O^6^ methylguanine-DNA methyltransferase (MGMT) is an enzyme that is able to transfer the methyl-adduct from O^6^ guanine to cysteine residue, which effectively repair the DNA alteration in tumor cells prior to replication [53]. Therefore, the presence of MGMT is considered to be the main reason for the resistance of tumor cells to alkylating agents. Li et al. have previously demonstrated that TMZ could induce the transcription of MGMT in glioma cells [54]. Some MGMT inhibitors, such as PaTrin-2, O^6^-benzylguanine, and streptozotocin, were analyzed in a combination treatment with TMZ, resulting in less activity of MGMT but with serious drug reactions or systematic toxicity [54–56]. Therefore, there is an urgent need to develop agents which combined with TMZ can inhibit the activity of MGMT and reduce the TMZ resistance. In the present study, PP7 was demonstrated to be efficient in a combination treatment with TMZ, by suppressing the expression of MGMT induced by TMZ treatment alone, and this effect may also through the modulating of AKT signaling, as AKT pathway was proven to be involved in the transcriptional regulation of MGMT [54, 57]. Our data suggested that the combination of PP7 with TMZ can be considered as an effective treatment for gliomas, but further works are still needed to prove the antiglioma effect of PP7 *in vivo*.

**Figure 8:**
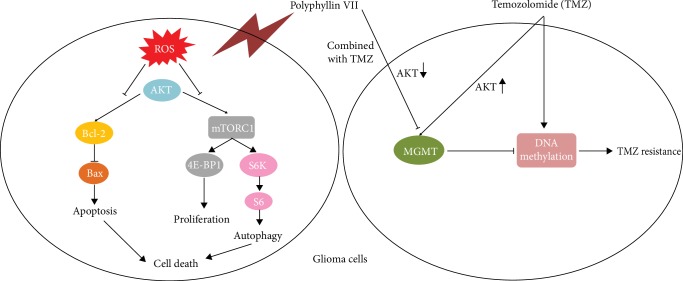
Schematic illustration of signals involved in cytotoxicity induced by PP7 (left) and by combination of PP7 with TMZ (right).

In conclusion, we provide for the first-time evidence that PP7 capably induces cell death in glioma cells, through the induction of apoptosis and autophagic cell death via ROS-induced AKT/mTORC1 activity inhibition (Figure 8, left). Moreover, combined treatments of PP7 with TMZ resulted in more effective cytotoxicity and decreased MGMT expression, which can be foreseen as a strategy to attenuate the ability of glioma cells to repair the TMZ-induced DNA methylation and therefore reduce the resistance of TMZ (Figure 8, right). Thus, our results suggest a novel therapeutic strategy based on PP7 treatment in gliomas. However, limitations are still present. First, it is unclear how PP7 stimulates the generation of ROS in glioma cells. Then, further evidence needs to be provided to understand whether the combined usage of PP7 and TMZ is effective in other glioma cell lines.

## Data Availability

The data used to support the findings of this study are available from the corresponding author upon request.
